# Supplementary material for: Cryopreservation method for Drosophila melanogaster embryos
Source: Nat Commun. 2021 Apr 23;12:2412. doi: 10.1038/s41467-021-22694-z (PMC8065140; doi:10.1038/s41467-021-22694-z)
Supplement: Supplementary file 1 — Supplementary Information [file 41467_2021_22694_MOESM1_ESM.pdf]

Supplementary Information for

Cryopreservation method for *Drosophila melanogaster* embryos

Li Zhan[1,2], Min-gang Li[3], Thomas Hays[3], John Bischof[1,2,4]

[1] Department of Mechanical Engineering, University of Minnesota, Minneapolis, MN, USA
[2] Center for Advanced Technologies for the Preservation of Biological Systems (ATP-Bio), University of Minnesota, Minneapolis, MN, USA
[3] Department of Genetics, Cell Biology and Development, University of Minnesota, Minneapolis, MN, USA
[4] Department of Biomedical Engineering, University of Minnesota, Minneapolis, MN, USA

Correspondence should be addressed to T.H. (email: haysx001@umn.edu) and J.B. (email: bischof@umn.edu).

This file includes:

Supplementary Figs. 1 to 29
Supplementary Tables 1 to 4

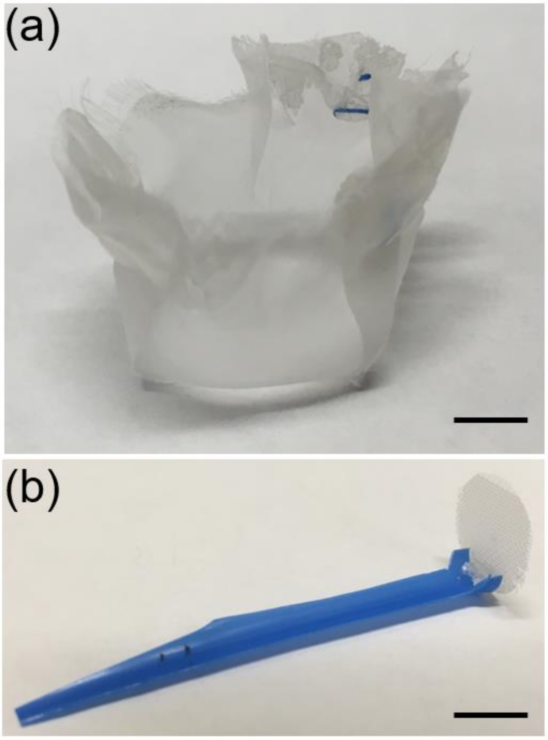

Supplementary Fig. 1. Simple tools used in this study. a-b, In this study, a simple nylon mesh basket (a) was used for embryo permeabilization. A cryomesh (b) and liquid nitrogen were used for vitrification. Scale bar is 1 cm. In comparison, specialized tools were required for embryo permeabilization and slush nitrogen preparation for vitrification in previous publication.[1]

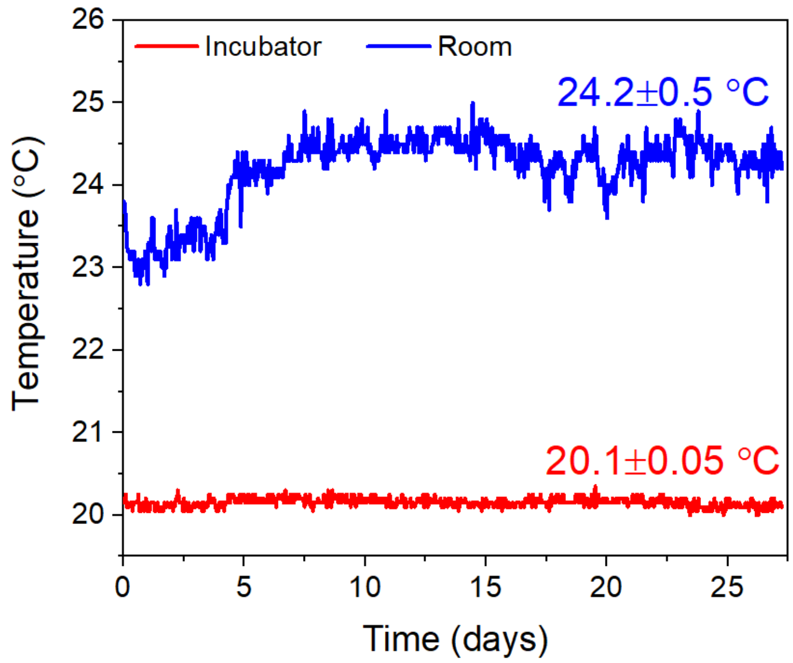

Supplementary Fig. 2. Temperature recording inside the incubator vs. room environment (i.e., lab). The incubator temperature was set to 20 °C to provide robust control of the embryo age for cryopreservation. Fluctuation of the room temperature will lead to inconsistent embryo age therefore inconsistent cryopreservation outcomes as the embryo developmental rate is temperature sensitive.

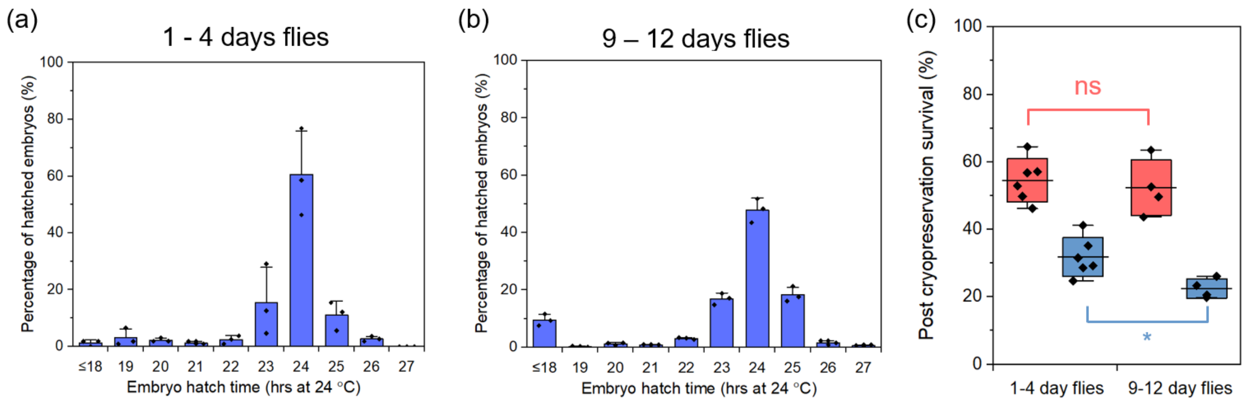

Supplementary Fig. 3. The age of the flies (strain M2) used for embryo collection impacts cryopreservation outcome. a, Embryo hatch frequency using 1 – 4 day old flies for embryo collection. **b,** Embryo hatch frequency using 9 – 12 day old flies for embryo collection. **c,** Comparison of post cryopreservation survival using flies of different ages for embryo collection. p value for hatch rate is 0.651, for adult rate is 0.018. Two-sided multivariate analysis of variance (MANOVA) and Tukey's post hoc were used for statistical analysis. ns, $p > 0.05$; * $p \leq 0.05$. Box and horizontal line represent standard deviation and mean respectively, whiskers represent max and min. Error bars represents standard deviation in (a-b). n=3 independent replicates for (a-b), n= 4 or 6 independent replicates for (c). In (c), red boxes present embryo hatch rate (i.e., embryo to larvae) and blue boxes represent adult rate (i.e., resulting larvae to adults). For 1-4 days flies, 1695 embryos were pooled over n=6 independent replicates; for 9-12 day flies, 1266 embryos were pooled over n=4 independent replicates. The same labeling applies to Figs. S4, S6, S7, S8, S9, S14, S15, S16, S17, S18, S19, S20, S21, S22, S23,S24, S25, S27.

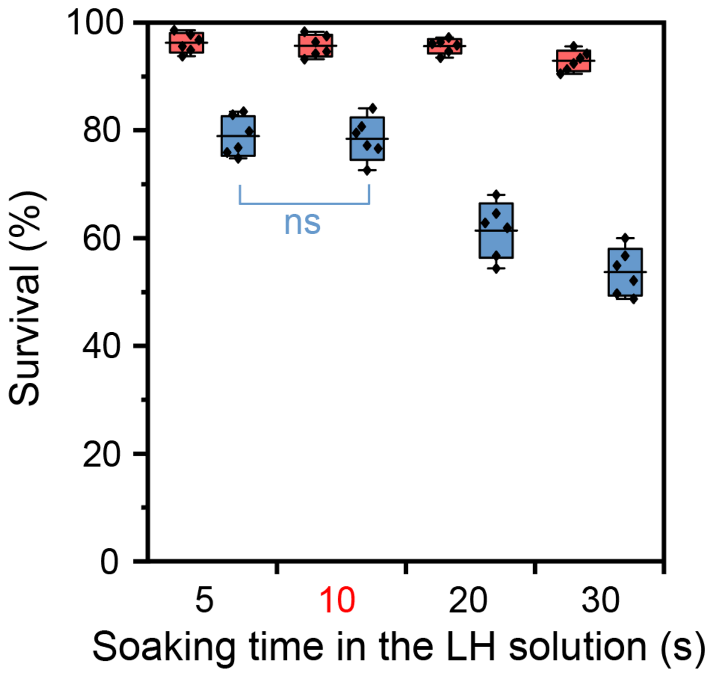

Supplementary Fig. 4. Survival of strain M2 after different soaking time in the LH solution. The 10s soaking time in the LH solution was selected to permeabilize the embryos after dechorionation. Box and horizontal line represent standard deviation and mean respectively; whiskers represent max and min. n=6 independent replicates. For 5s, 2140 embryos were pooled over n=6 independent replicates; for 10s, 1925 embryos were pooled over n=6 independent replicates; for 20s, 1922 embryos were pooled over n=6 independent replicates; for 30s, 1762 embryos were pooled over n=6 independent replicates. Two-sided multivariate analysis of variance (MANOVA) and Tukey's post hoc were used for statistical analysis. ns, $p > 0.05$.

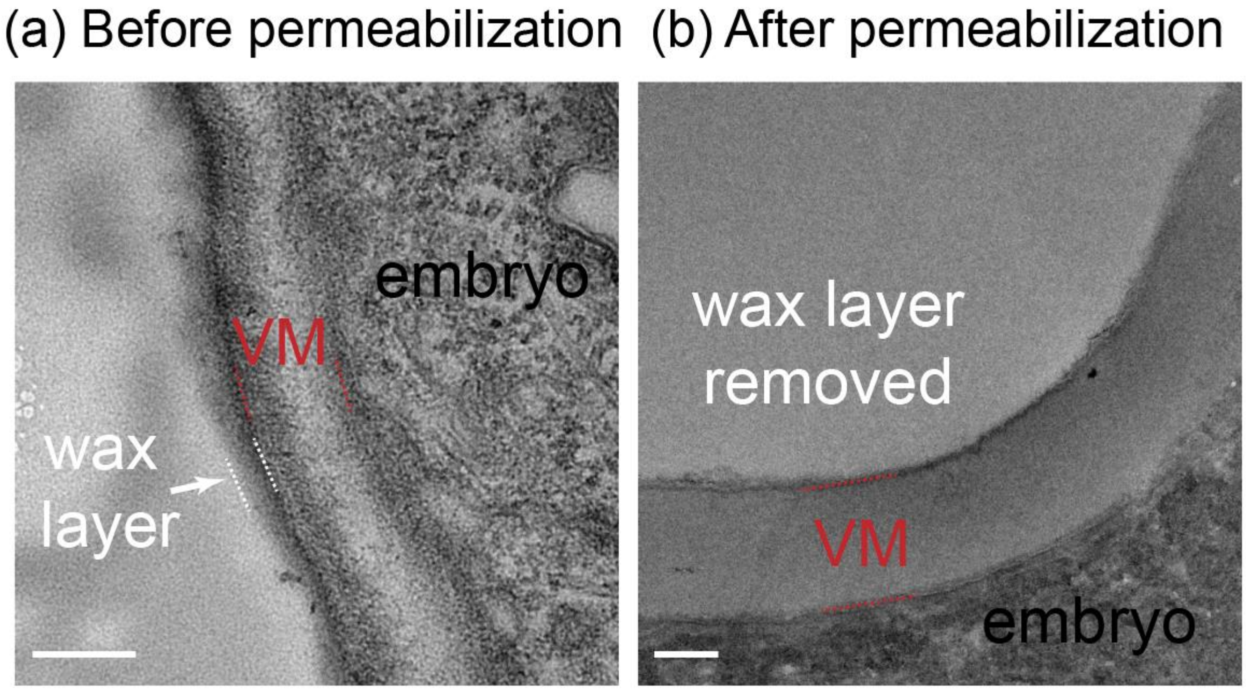

Supplementary Fig. 5. EM images of *w[1118]* embryos before and after permeabilization. a, Before permeabilization, a wax layer can be identified outside the vitelline membrane (VM) White and red dashed lines indicate the boundaries of wax layer and VM, respectively. **b,** After permeabilization, wax layer was removed. Three experiments were repeated independently with similar results for (a-b). Scale bar is 200 nm.

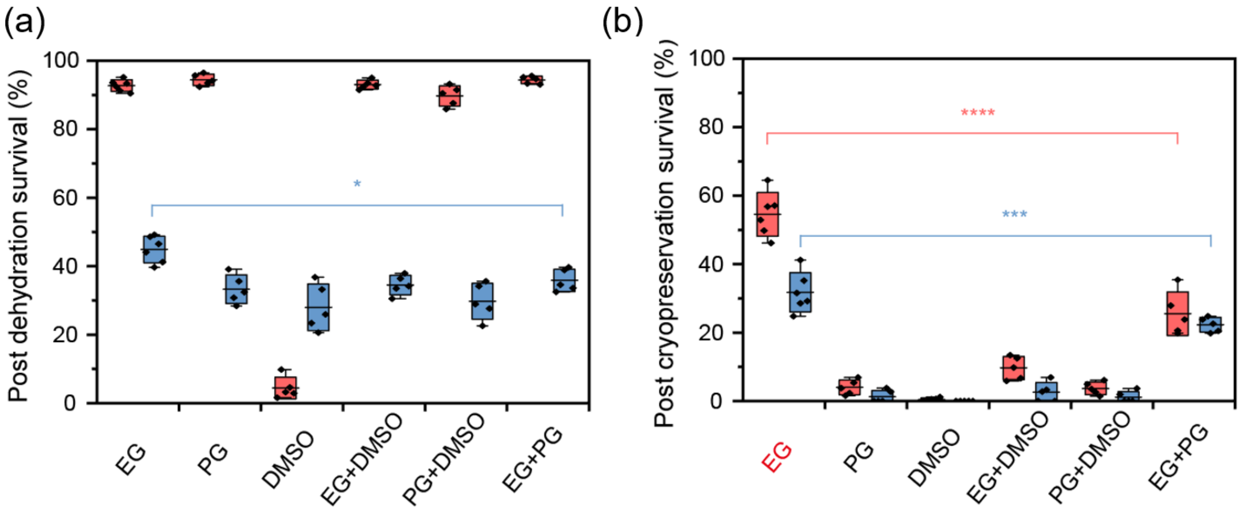

Supplementary Fig. 6. Post dehydration and post cryopreservation survival of strain M2 using different permeable CPAs and cocktails. a, Embryos were treated with dehydration CPA followed by unloading steps (i.e., no cryopreservation) to evaluate the CPA toxicity. **b,** Post cryopreservation survival using different CPAs and cocktails. The 39 wt % CPA (or cocktail) + 9 wt% sorbitol was used. Cocktails were prepared using equal parts of individual CPAs. For example, EG + DMSO represents 19.5 wt% EG + 19.5 wt% DMSO + 9 wt% sorbitol. EG was eventually selected. Box and horizontal line represent standard deviation and mean respectively; whiskers represent max and min. n=5 or 6 independent replicates in (a-b). In (a), for EG, 1950 embryos were pooled over n=6 independent replicates; for PG, 1704 embryos were pooled over n=5 independent replicates; for DMSO, 1523 embryos were pooled over n=5 independent replicates; for EG+DMSO, 1569 embryos were pooled over n=5 independent replicates; for PG+DMSO, 1501 embryos were pooled over n=5 independent replicates; for EG+DMSO, 1475 embryos were pooled over n=5 independent replicates. In (b), for EG, 1695 embryos were pooled over n=6 independent replicates; for PG, 1536 embryos were pooled over n=5 independent replicates; for DMSO, 1477 embryos were pooled over n=5 independent replicates; for EG+DMSO, 1471 embryos were pooled over n=5 independent replicates; for PG+DMSO, 1614 embryos were pooled over n=5 independent replicates; for EG+DMSO, 1600 embryos were pooled over n=5 independent replicates. Two-sided multivariate analysis of variance (MANOVA) and Tukey's post hoc were used for statistical analysis. * $p \leq 0.01$; *** $p \leq 0.001$; **** $p \leq 0.0001$.

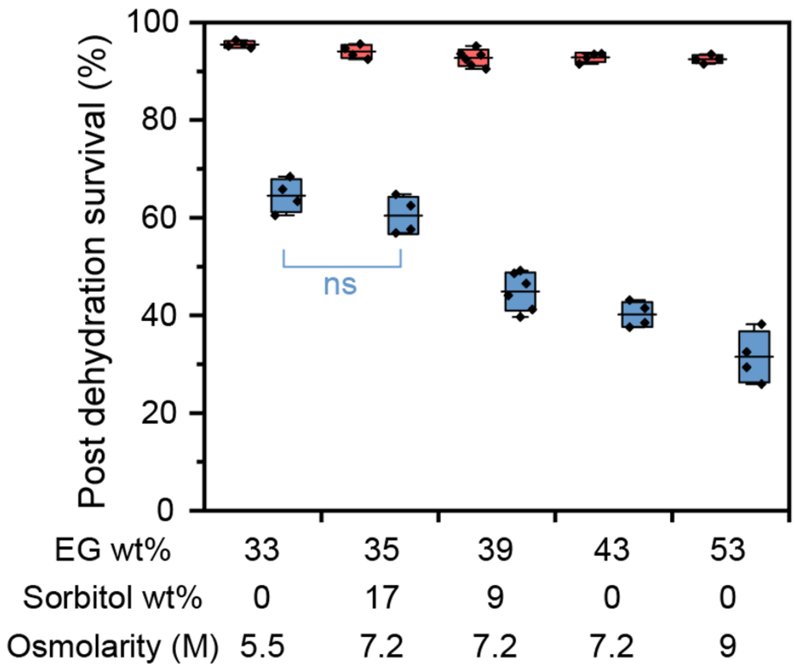

Supplementary Fig. 7. Post dehydration survival of strain M2 using different CPAs. Different combinations of EG and sorbitol were tested to evaluate the post dehydration survival. Box and horizontal line represent standard deviation and mean respectively; whiskers represent max and min. n=4 or 6 independent replicates. For 33% EG, 1326 embryos were pooled over n=4 independent replicates; for 35% EG + 17 % sorbitol, 1209 embryos were pooled over n=4 independent replicates; for 39% EG + 9% sorbitol, 1878 embryos were pooled over n=6 independent replicates; for 43% EG, 1247 embryos were pooled over n=4 independent replicates; for 53% EG, 1376 embryos were pooled over n=4 independent replicates. Two-sided multivariate analysis of variance (MANOVA) and Tukey's post hoc were used for statistical analysis. ns, $p > 0.05$.

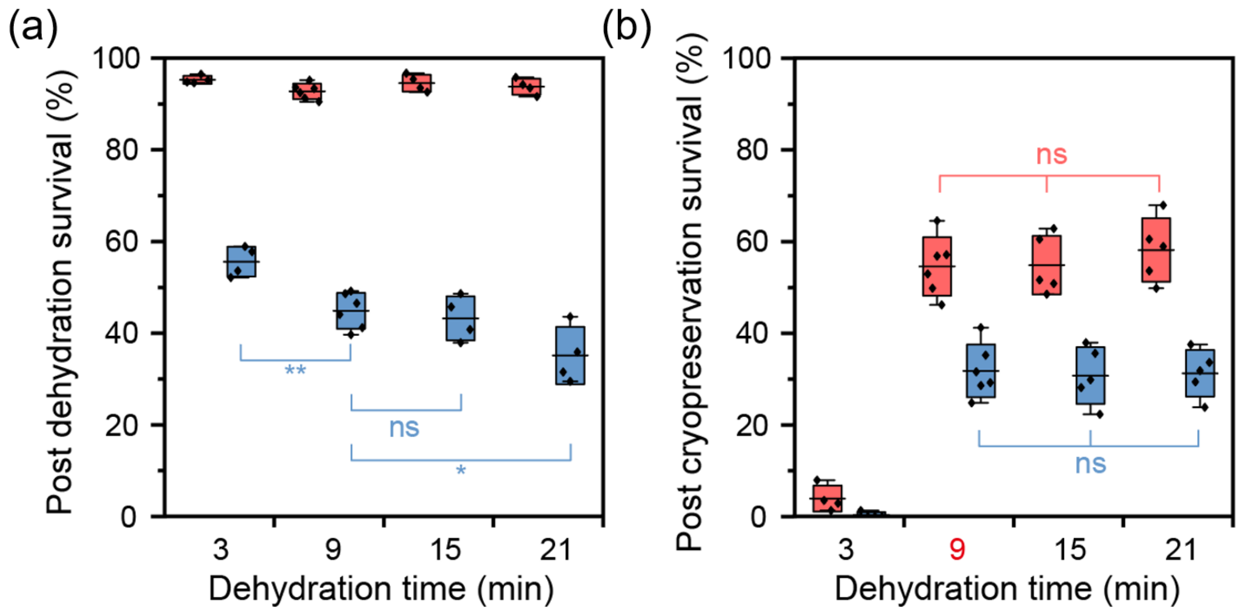

Supplementary Fig. 8. Post dehydration and post cryopreservation survival of strain M2 using different dehydration time. a, Post dehydration survival. **b,** Post cryopreservation survival. The dehydration CPA is 39 wt % EG + 9 wt% sorbitol. Dehydration time of 9 min was selected. Box and horizontal line represent standard deviation and mean respectively; whiskers represent max and min. n=4 or 6 independent replicates for (a-b). In (a), for 3 min, 1335 embryos were pooled over n=4 independent replicates; for 9 min, 1757 embryos were pooled over n=6 independent replicates; for 15 min, 1330 embryos were pooled over n=4 independent replicates; for 21 min, 1099 embryos were pooled over n=4 independent replicates. In (b), for 3 min, 1175 embryos were pooled over n=4 independent replicates; for 9 min, 1695 embryos were pooled over n=6 independent replicates; for 15 min, 1616 embryos were pooled over n=5 independent replicates; for 21 min, 1671 embryos were pooled over n=5 independent replicates. Two-sided multivariate analysis of variance (MANOVA) and Tukey's post hoc were used for statistical analysis. ns, $p > 0.05$; * $p \leq 0.05$; ** $p \leq 0.01$.

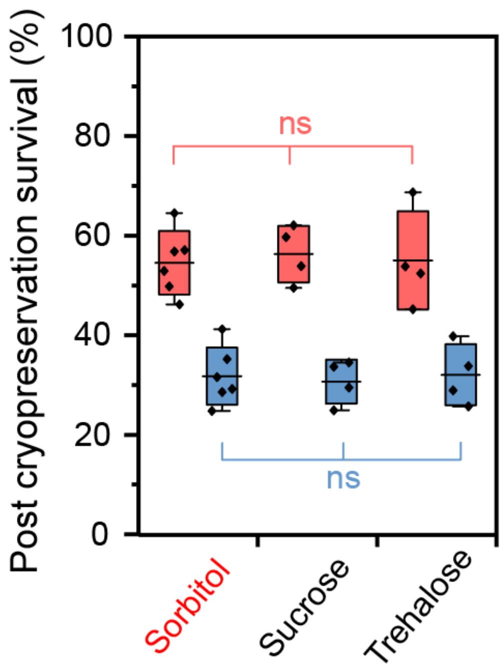

Supplementary Fig. 9. Post cryopreservation survival of strain M2 using different sugar in the dehydration CPA. Sorbitol, sucrose and trehalose provides similar survival. Sorbitol was selected due to lower cost. Box and horizontal line represent standard deviation and mean respectively; whiskers represent max and min. n=4 or 6 independent replicates. For sorbitol, 1695 embryos were pooled over n=6 independent replicates; for sucrose, 1304 embryos were pooled over n=4 independent replicates; for trehalose, 1417 embryos were pooled over n=4 independent replicates. Two-sided multivariate analysis of variance (MANOVA) and Tukey's post hoc were used for statistical analysis. ns, $p > 0.05$.

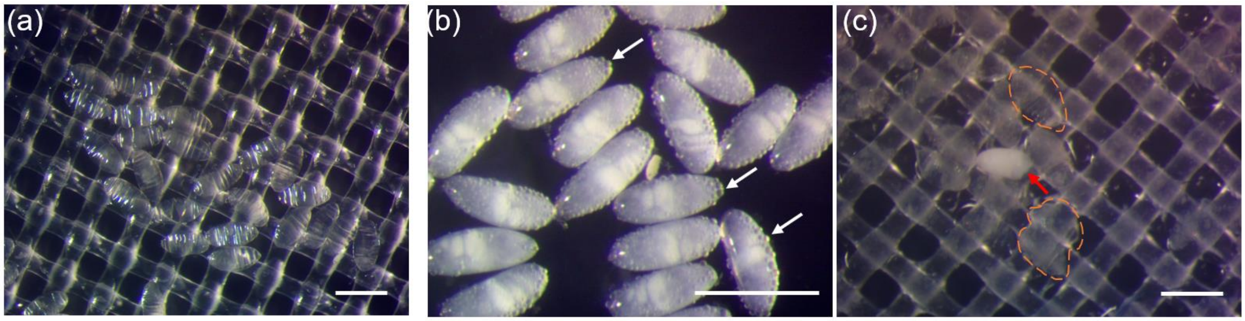

Supplementary Fig. 10. Images of embryos during cryopreservation. a, Dehydrated embryos on the cryomesh after removing CPA solution. **b,** Liquid exchange across the vitelline membrane during CPA unloading. Floating embryos show tiny liquid droplets leaving the embryo surface. The vitelline membrane helps to maintain the integrity of the embryo during CPA unloading process. **c,** Dehydrated embryos in liquid nitrogen. Embryos circled in orange are vitrified embryos showing transparent appearance. Red arrow indicates a crystallized embryo (i.e., failure). Scale bar is 500 µm. Five experiments were repeated independently with similar results for (a-c).

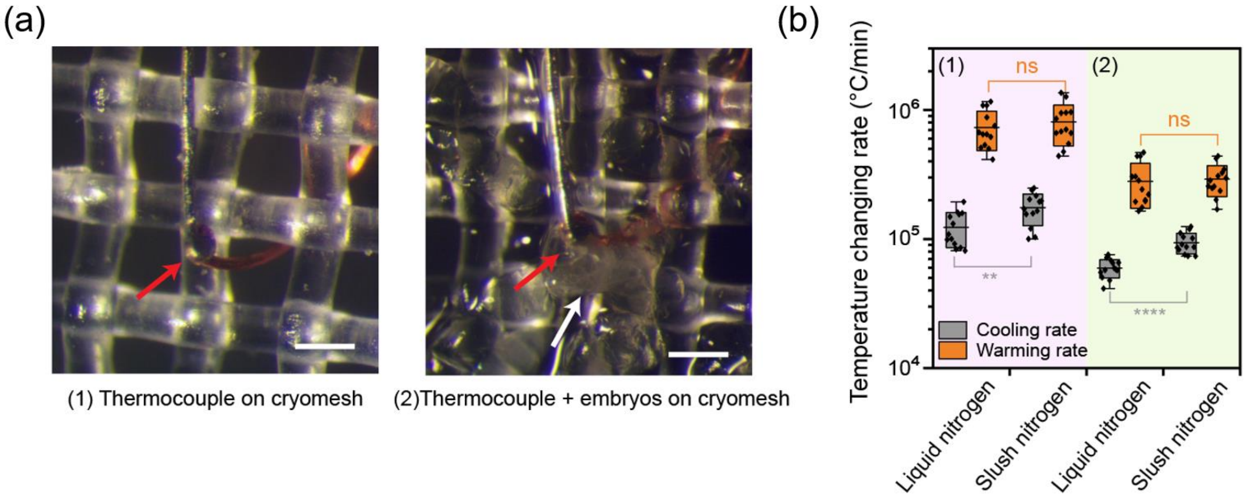

Supplementary Fig. 11. Measurement of cooling and warming rate in liquid nitrogen and slush nitrogen. a, Images of a thermocouple for cooling and warming rate measurement. (1) thermocouple alone on the cryomesh and (2) thermocouple in contact with dehydrated embryos on the cryomesh. Red arrows indicate the thermocouple junction. White arrow indicates the dehydrated embryos. Five experiments were repeated independently with similar results. **b,** Measured cooling and warming rate using liquid nitrogen and slush nitrogen in two settings described in (a). Box and horizontal line represent standard deviation and mean respectively; whiskers represent max and min. n=13 independent replicates for (b). Two-sided multivariate analysis of variance (MANOVA) and Tukey's post hoc were used for statistical analysis. ns: $p>0.05$; ** $p \leq 0.01$; **** $p \leq 0.0001$.

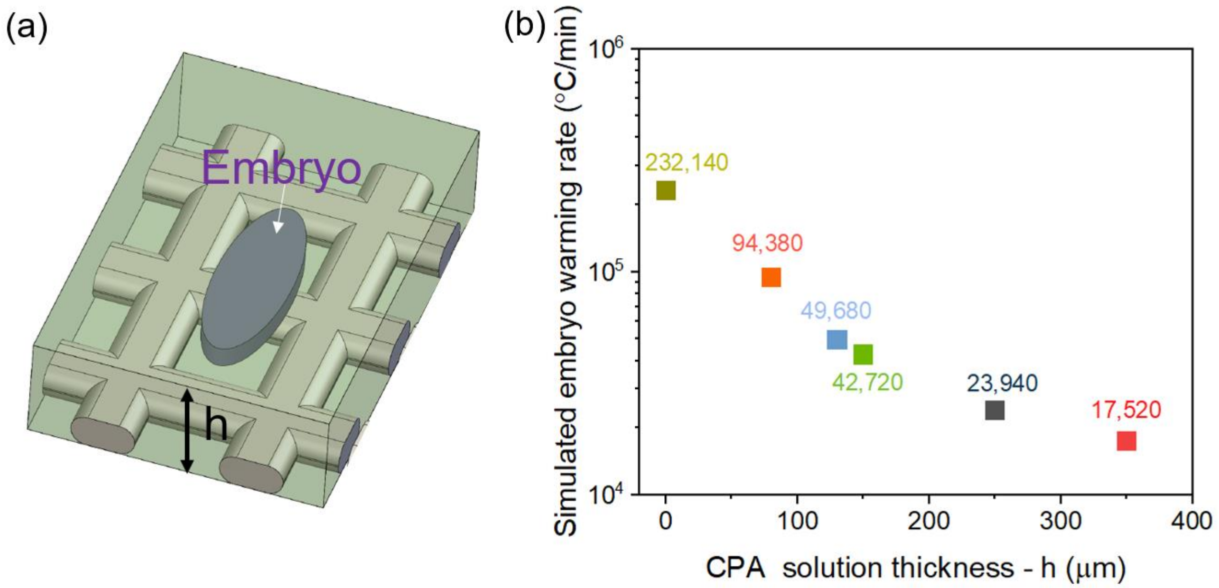

Supplementary Fig. 12. Simulated warming rate of the embryo with different thickness of CPA solution around. a, Embryo located on the cryomesh was surrounded by CPA solution with a thickness of *h*. **b,** Simulated warming rate at the center of the embryo decreases with increasing CPA layer thickness.

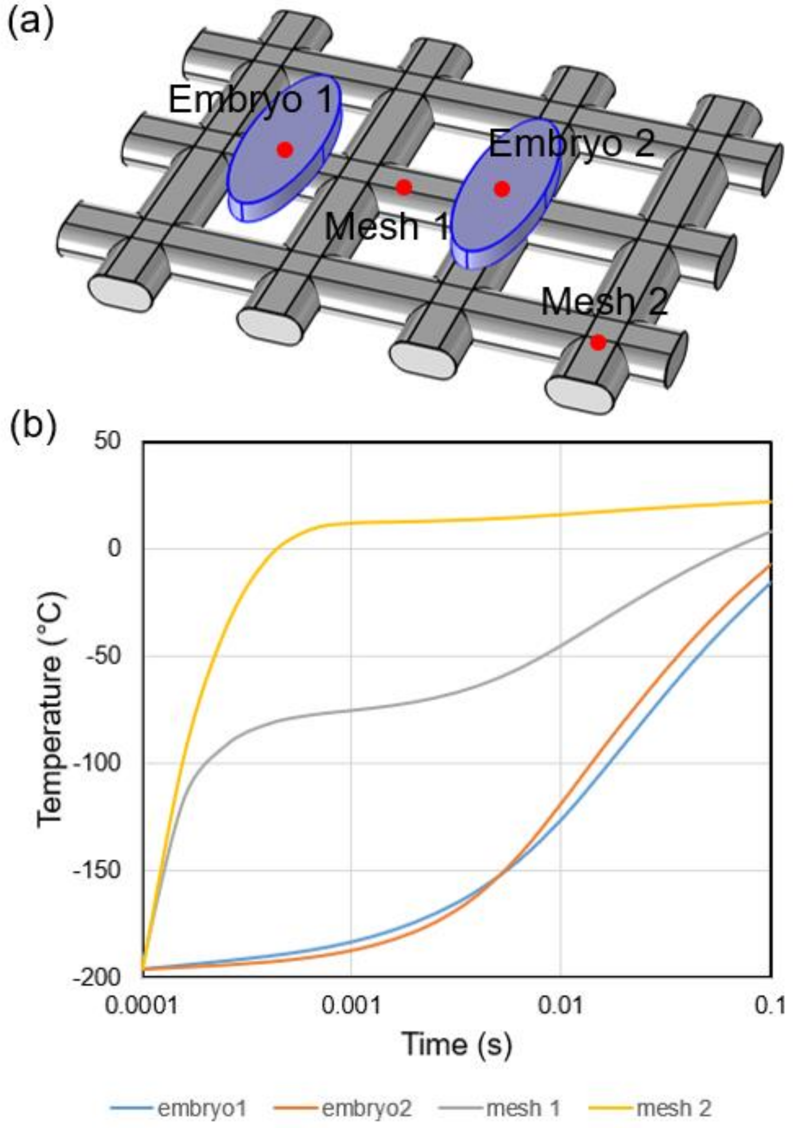

Supplementary Fig. 13. Simulated temperature profile of embryos and nylon mesh during rewarming . a, 4 points were selected for evaluation during modeling including the center point of embryo 1, the center point of embryo 2, the center point of nylon between embryo 1 and embryo 2, and the center point of nylon far away from embryos. **b,** Simulated temperature profile at the 4 points from a. As the Nylon (mesh 1 and mesh 2) rewarmed faster they are able to diffuse heat towards the embryos thereby enhancing their warming rates.

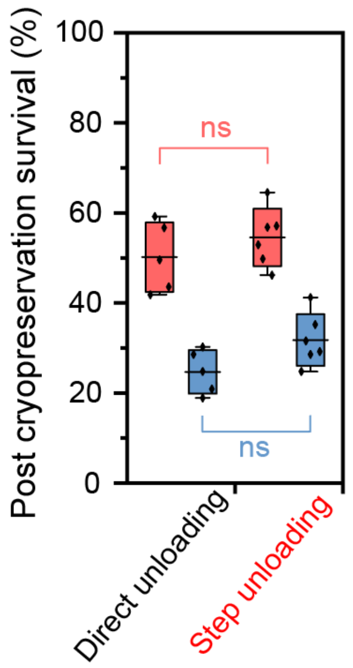

Supplementary Fig. 14. Post cryopreservation survival of strain M2 using different CPA unloading methods. Step unloading was selected owing to slightly higher adult rate. Box and horizontal line represent standard deviation and mean respectively; whiskers represent max and min. n=5 or 6 independent replicates. For direction unloading, 1652embryos were pooled over n=5 independent replicates; for step unloading, 1695 embryos were pooled over n=6 independent replicates. Two-sided multivariate analysis of variance (MANOVA) and Tukey's post hoc were used for statistical analysis. ns, $p > 0.05$.

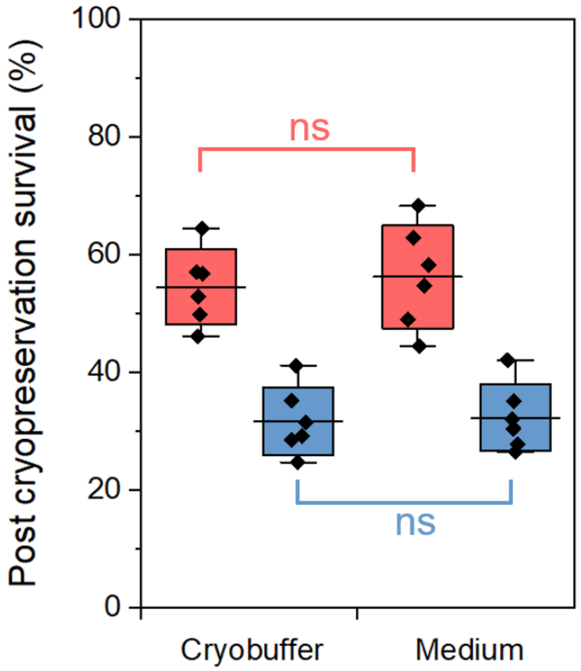

Supplementary Fig. 15. Post cryopreservation survival of strain M2 using cryobuffer vs. Schneider medium to prepare CPA solutions and unloading solutions. No significant (ns) difference was observed between these cases. *p* value for hatch rate is 0.704, for adult rate is 0.86. The use of cryobuffer will greatly reduce the cost of cryopreservation. Box and horizontal line represent standard deviation and mean respectively; whiskers represent max and min. n=6 independent replicates. For cryobuffer, 1695 embryos were pooled over n=6 independent replicates; for medium, 1674 embryos were pooled over n=6 independent replicates. Two-sided multivariate analysis of variance (MANOVA) and Tukey's post hoc were used for statistical analysis. ns, $p > 0.05$.

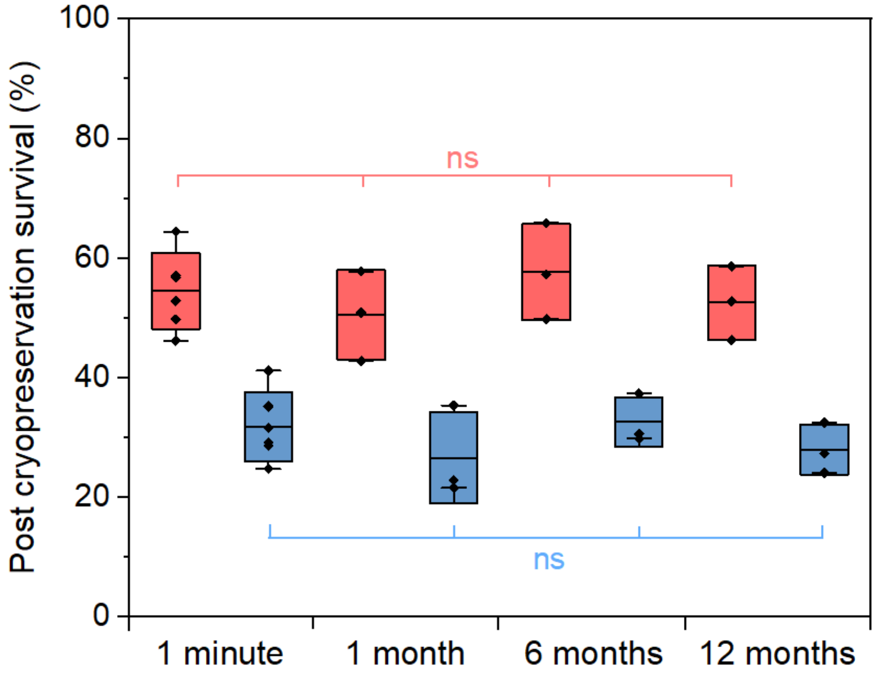

Supplementary Fig. 16. Post cryopreservation survival of strain M2 after different storage in liquid nitrogen. Box and horizontal line represent standard deviation and mean respectively; whiskers represent max and min. n= 3 or 6 independent replicates. For 1 minute, 1695 embryos were pooled over n=6 independent replicates; for 1 month, 844 embryos were pooled over n=3 independent replicates; for 6 months, 964 embryos were pooled over n=3 independent replicates; for 12 months, 946 embryos were pooled over n=3 independent replicates. Two-sided multivariate analysis of variance (MANOVA) and Tukey's post hoc were use d for statistical analysis. ns, $p > 0.05$.

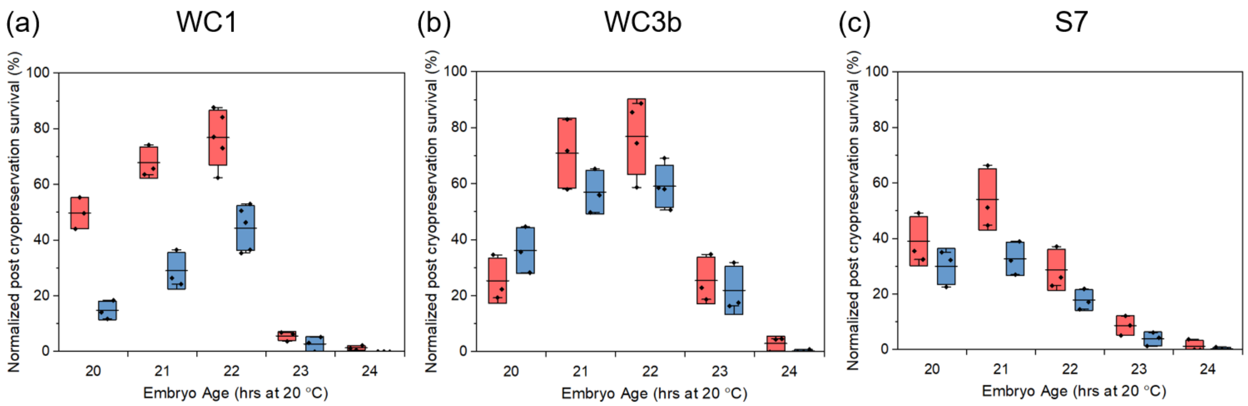

Supplementary Fig. 17. The effect of embryo age on normalized post cryopreservation survival using various strains. a-c, strain WC1, WC3b and S7. Box and horizontal line represent standard deviation and mean respectively; whiskers represent max and min. n= 3 or 5 independent replicates for (a-c). In (a), for the embryo age of 20 hrs, 912 embryos were pooled over n=3 independent replicates; for the embryo age of 21 hrs, 872 embryos were pooled over n=3 independent replicates; for the embryo age of 22 hrs, 1439 embryos were pooled over n=5 independent replicates; for the embryo age of 23 hrs, 815 embryos were pooled over n=3 independent replicates; for the embryo age of 24 hrs, 834 embryos were pooled over n=3 independent replicates. In (b), for the embryo age of 20 hrs, 832 embryos were pooled over n=3 independent replicates; for the embryo age of 21 hrs, 989 embryos were pooled over n=3 independent replicates; for the embryo age of 22 hrs, 1267 embryos were pooled over n=4 independent replicates; for the embryo age of 23 hrs, 864 embryos were pooled over n=3 independent replicates; for the embryo age of 24 hrs, 844 embryos were pooled over n=3 independent replicates. In (c), for the embryo age of 20 hrs, 934 embryos were pooled over n=3 independent replicates; for the embryo age of 21 hrs, 1102 embryos were pooled over n=3 independent replicates; for the embryo age of 22 hrs, 936 embryos were pooled over n=3 independent replicates; for the embryo age of 23 hrs, 819 embryos were pooled over n=3 independent replicates; for the embryo age of 24 hrs, 885 embryos were pooled over n=3 independent replicates.

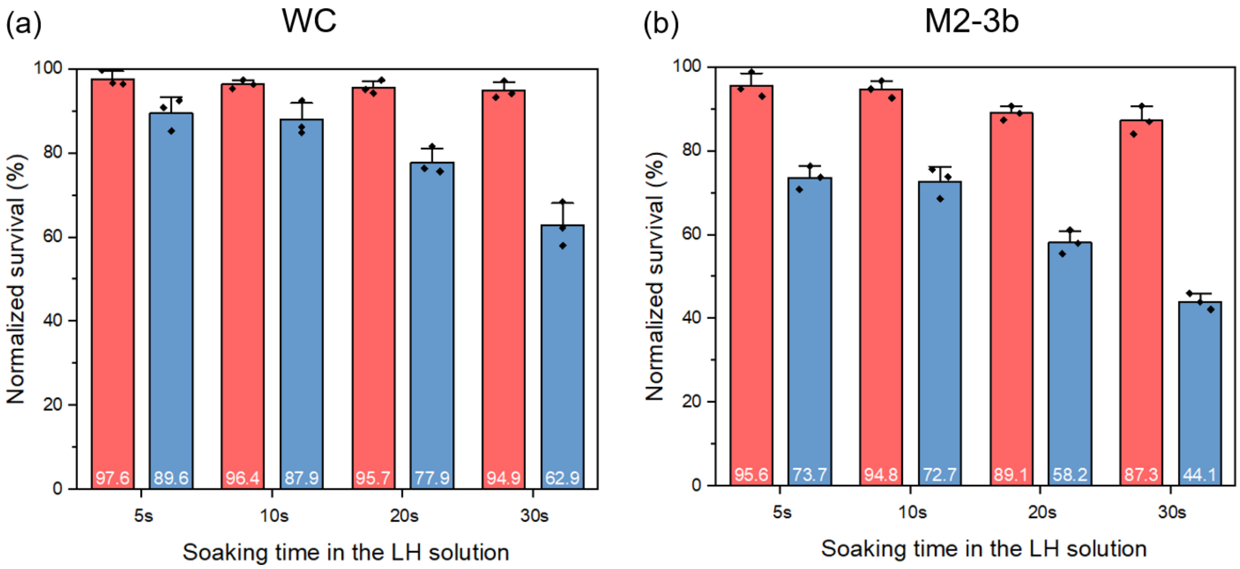

Supplementary Fig. 18. The effect of soaking time in the permeabilization solution (LH solution) on normalized survival using various strains. a-b, strain WC, M2-3b. n=3 independent replicates for (a-b). In (a), for 5s, 1304 embryos were pooled over n=3 independent replicates; for 10s, 1246 embryos were pooled over n=3 independent replicates; for 20s, 1173 embryos were pooled over n=3 independent replicates; for 30s, 1232 embryos were pooled over n=3 independent replicates. In (b), for 5s, 857 embryos were pooled over n=3 independent replicates; for 10s, 1060 embryos were pooled over n=3 independent replicates; for 20s, 998 embryos were pooled over n=3 independent replicates; for 30s, 1038 embryos were pooled over n=3 independent replicates. Error bars represent standard deviation.

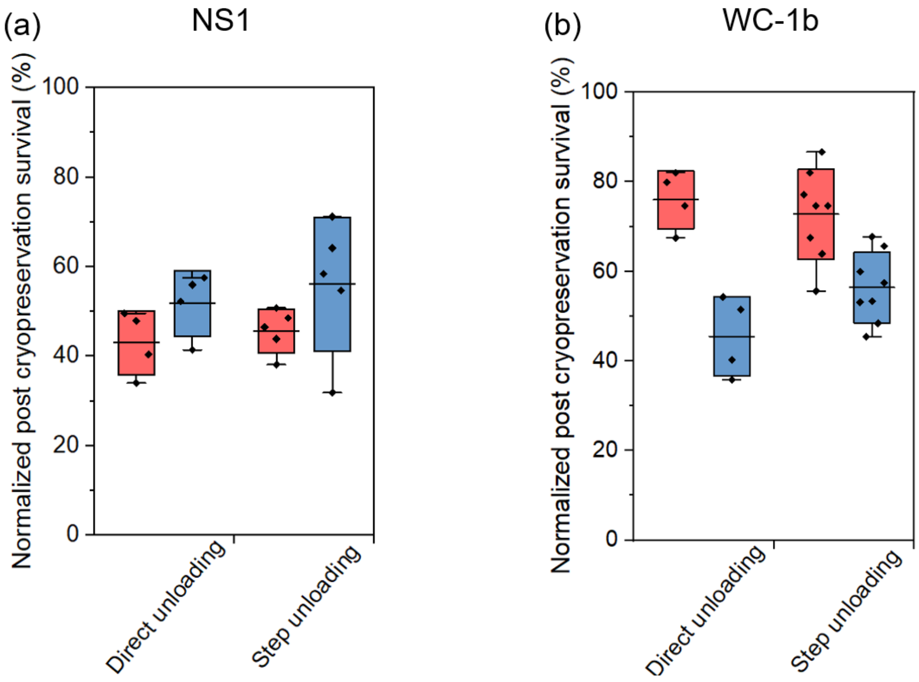

Supplementary Fig. 19. The effect of unloading methods on normalized post cryopreservation survival using various strains. a-b, strain NS1, WC-1b. Box and horizontal line represent standard deviation and mean respectively; whiskers represent max and min. n=4 or 5 independent replicates for (a), n=4 or 8 independent replicates for (b). In (a), for direction unloading, 1156 embryos were pooled over n=4 independent replicates; for step unloading, 1478 embryos were pooled over n=5 independent replicates. In (b), for direction unloading, 1446 embryos were pooled over n=4 independent replicates; for step unloading, 2519 embryos were pooled over n=8 independent replicates.

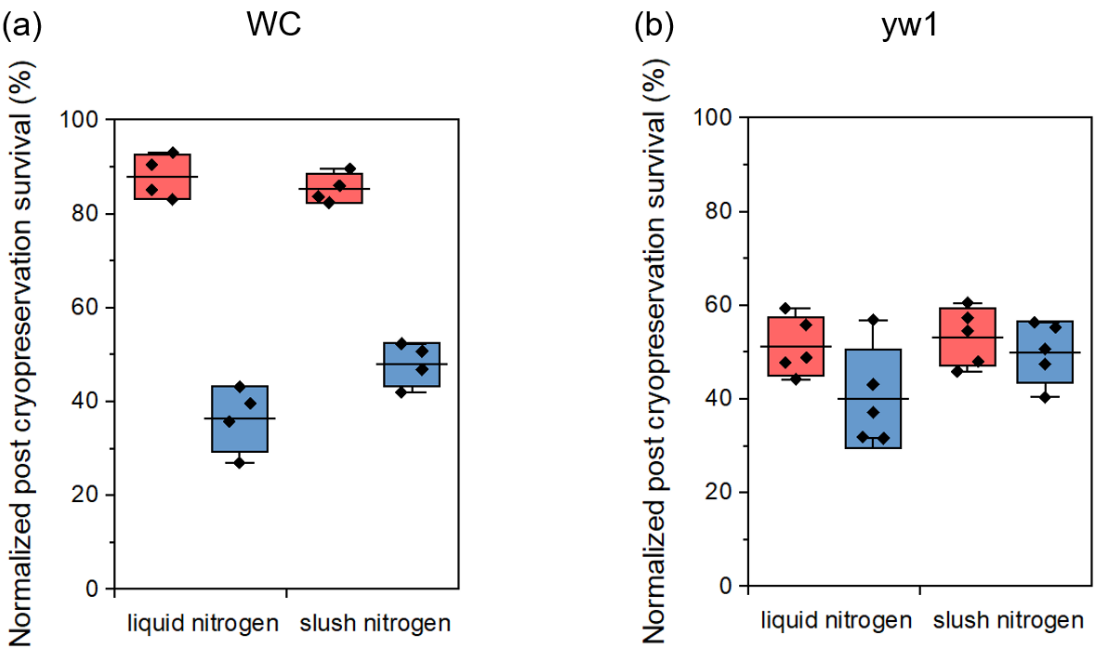

Supplementary Fig. 20. The effect of cryogen on normalized post cryopreservation survival using various strains. a-b, strain WC, yw1. Box and horizontal line represent standard deviation and mean respectively; whiskers represent max and min. n=4 independent replicates for (a), n=5 independent replicates for (b). In (a), for liquid nitrogen, 1874 embryos were pooled over n=4 independent replicates; for slush nitrogen, 1597 embryos were pooled over n=4 independent replicates. In (b), for liquid nitrogen, 1971 embryos were pooled over n=5 independent replicates; for slush nitrogen, 2063 embryos were pooled over n=5 independent replicates.

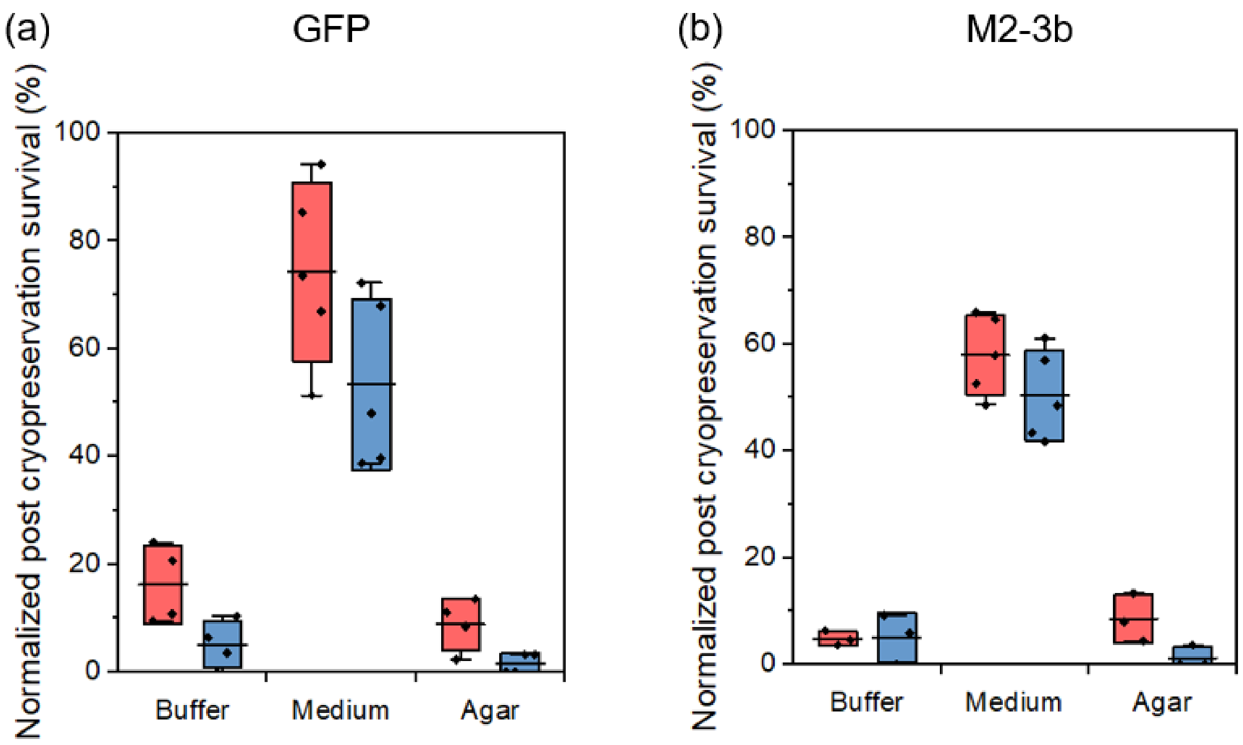

Supplementary Fig. 21. The effect of embryo culture methods on normalized post cryopreservation survival using various strains. a-b, strain GFP, M2-3b. Box and horizontal line represent standard deviation and mean respectively; whiskers represent max and min. n=4 or 5 independent replicates for (a). n=3 or 5 independent replicates for (b). In (a), for buffer, 1454 embryos were pooled over n=4 independent replicates; for medium, 1859 embryos were pooled over n=5 independent replicates; for agar, 1293 embryos were pooled over n=4 independent replicates. In (b), for buffer, 975 embryos were pooled over n=3 independent replicates; for medium, 1612 embryos were pooled over n=5 independent replicates; for agar, 1041 embryos were pooled over n=3 independent replicates.

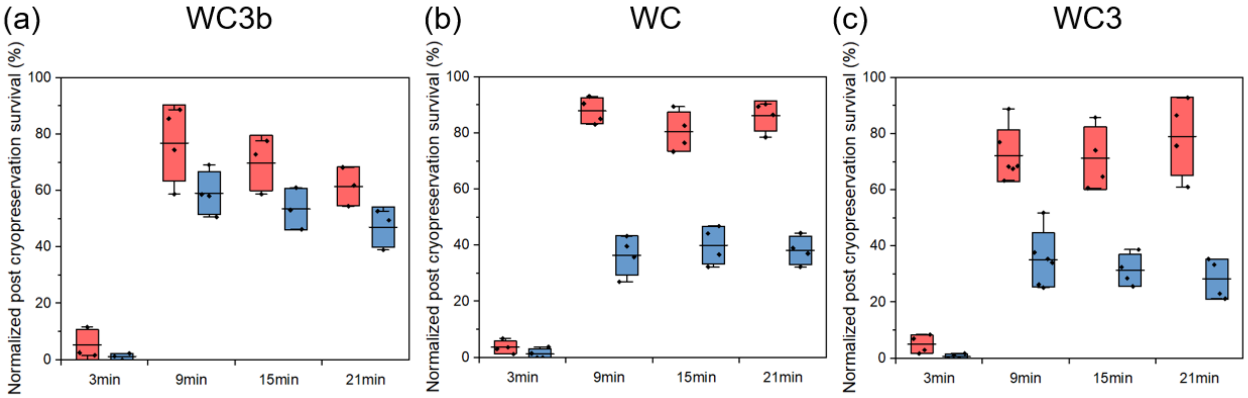

Supplementary Fig. 22. The effect of dehydration time on normalized post cryopreservation survival using various strains. a-c, strain WC3b, WC, WC3. 39 wt% EG + 9 wt% sorbitol was used. Box and horizontal line represent standard deviation and mean respectively; whiskers represent max and min. n=3 or 4 independent replicates for (a-c). In (a), for 3 min, 896 embryos were pooled over n=3 independent replicates; for 9 min, 1267 embryos were pooled over n=4 independent replicates; for 15 min, 977 embryos were pooled over n=3 independent replicates; for 21 min, 853 embryos were pooled over n=3 independent replicates. In (b), for 3 min, 1784 embryos were pooled over n=4 independent replicates; for 9 min, 1874 embryos were pooled over n=4 independent replicates; for 15 min, 1380 embryos were pooled over n=4 independent replicates; for 21 min, 1421 embryos were pooled over n=4 independent replicates. In (c), for 3 min, 1130 embryos were pooled over n=4 independent replicates; for 9 min, 1741 embryos were pooled over n=6 independent replicates; for 15 min, 1136 embryos were pooled over n=4 independent replicates; for 21 min, 1132 embryos were pooled over n=4 independent replicates.

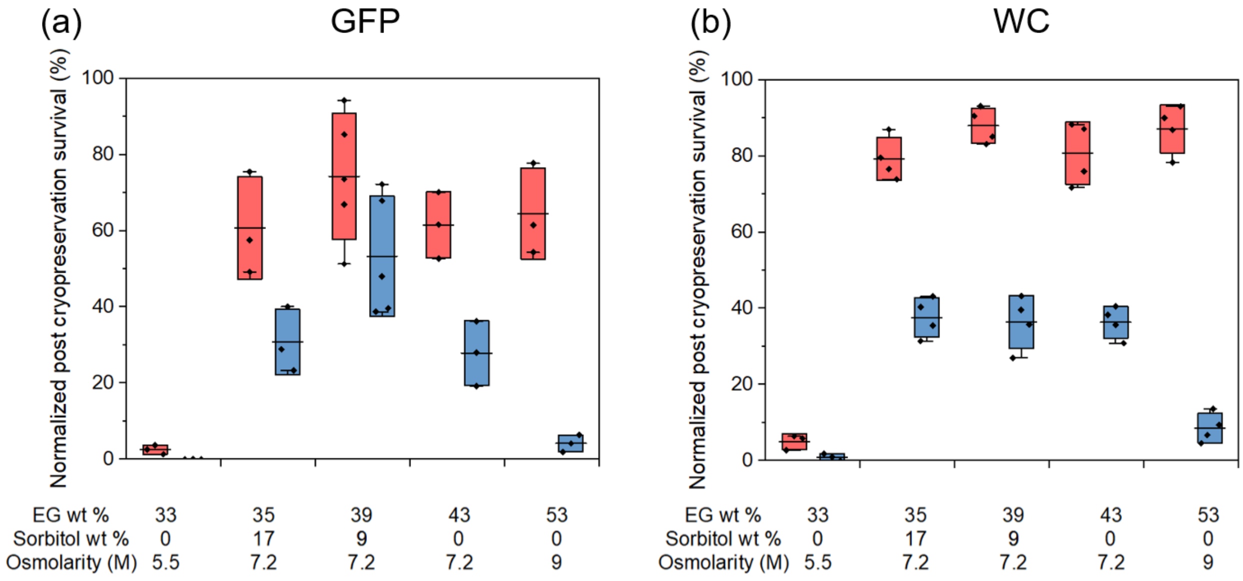

Supplementary Fig. 23. The effect of dehydration CPA on normalized post cryopreservation survival using various strains. a-b, strain GFP, WC. 9 min dehydration time was used. Box and horizontal line represent standard deviation and mean respectively; whiskers represent max and min. n=3 or 5 independent replicates for (a-b). In (b), for 33 wt% EG, 996 embryos were pooled over n=3 independent replicates; for 35 wt% EG + 17 wt% sorbitol, 1083 embryos were pooled over n=3 independent replicates; for 39 wt% EG + 9 wt% sorbitol, 1859 embryos were pooled over n=5 independent replicates; for 43 wt% EG, 963 embryos were pooled over n=3 independent replicates; for 53 wt% EG, 1074 embryos were pooled over n=3 independent replicates. In (b), for 33 wt% EG, 1300 embryos were pooled over n=3 independent replicates; for 35 wt% EG + 17 wt% sorbitol, 1077 embryos were pooled over n=3 independent replicates; for 39 wt% EG + 9 wt% sorbitol, 1874 embryos were pooled over n=4 independent replicates; for 43 wt% EG, 1673 embryos were pooled over n=4 independent replicates; for 53 wt% EG, 1385 embryos were pooled over n=4 independent replicates.

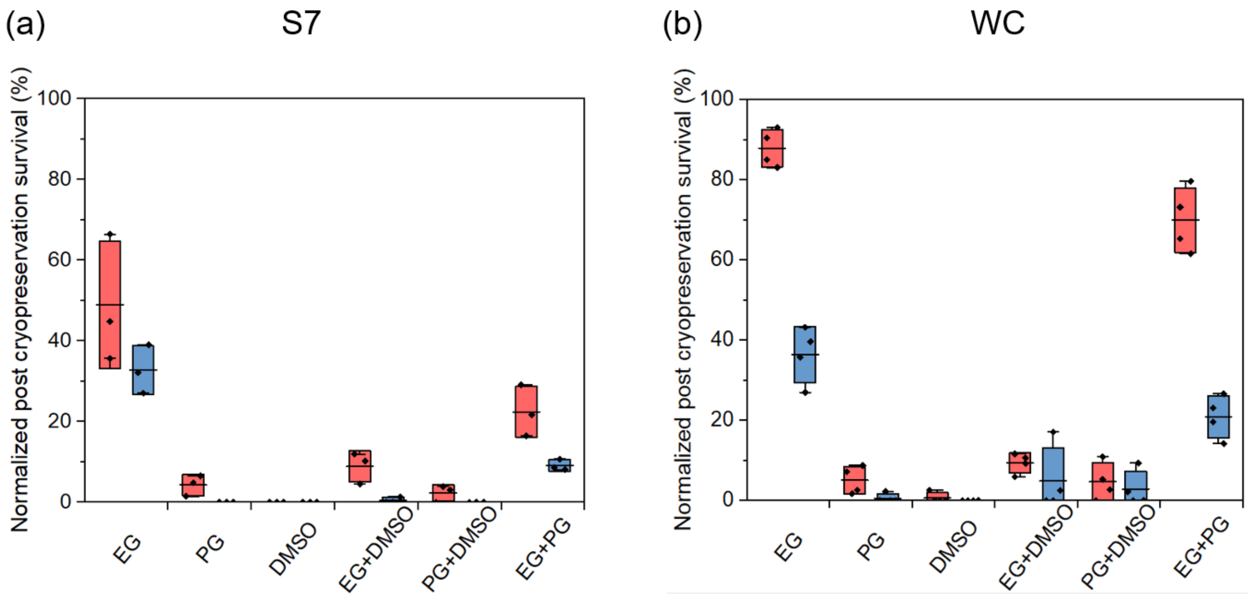

Supplementary Fig. 24. The effect of permeable CPA (or cocktail) on normalized post cryopreservation survival using various strains. a-b, strain S7, WC. 13 wt% CPA (or cocktail) was used for first step loading, 39 wt% CPA (or cocktail) + 9 wt% sorbitol was used for dehydration. 9 min dehydration time was used. Box and horizontal line represent standard deviation and mean respectively; whiskers represent max and min. n=3 or 4 independent replicates for (a-b). In (a), for EG, 1081 embryos were pooled over n=3 independent replicates; for PG, 1017 embryos were pooled over n=3 independent replicates; for DMSO, 945 embryos were pooled over n=3 independent replicates; for EG+DMSO, 938 embryos were pooled over n=3 independent replicates; for PG+DMSO, 967 embryos were pooled over n=3 independent replicates; for EG+DMSO, 947 embryos were pooled over n=3 independent replicates. In (b), for EG, 1874 embryos were pooled over n=4 independent replicates; for PG, 1771 embryos were pooled over n=4 independent replicates; for DMSO, 1638 embryos were pooled over n=4 independent replicates; for EG+DMSO, 1430 embryos were pooled over n=4 independent replicates; for PG+DMSO, 1590 embryos were pooled over n=4 independent replicates; for EG+DMSO, 1513 embryos were pooled over n=4 independent replicates.

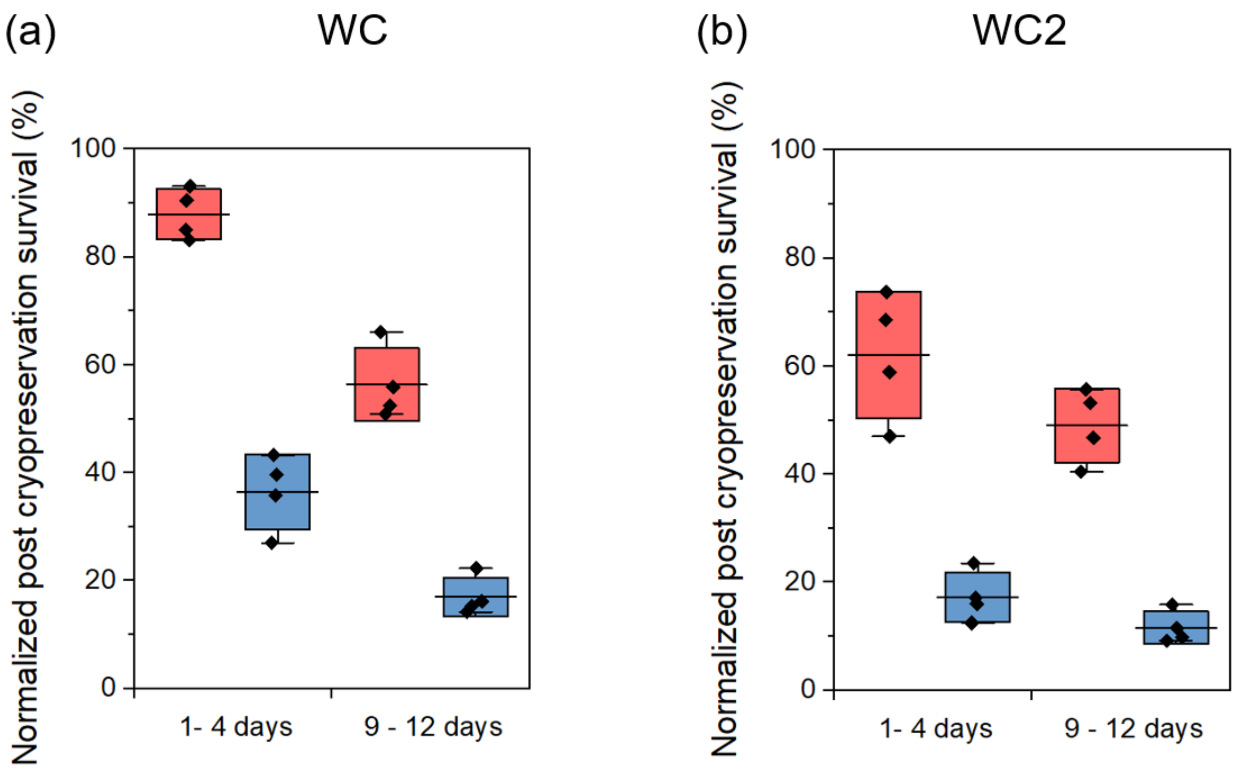

Supplementary Fig. 25. The age of the flies used for embryo collection impacts cryopreservation outcome using various strains. a-b, strain WC, WC2. Box and horizontal line represent standard deviation and mean respectively; whiskers represent max and min. n=3 or 4 independent replicates for (a-b). In (a), red boxes present embryo hatch rate (i.e., embryo to larvae) and blue boxes represent adult rate (i.e., resulting larvae to adults). For 1-4 days flies, 1874 embryos were pooled over n=4 independent replicates; for 9-12 day flies, 1513 embryos were pooled over n=4 independent replicates. In (b), red boxes present embryo hatch rate (i.e., embryo to larvae) and blue boxes represent adult rate (i.e., resulting larvae to adults). For 1-4 days flies, 1664 embryos were pooled over n=4 independent replicates; for 9-12 day flies, 1457 embryos were pooled over n=4 independent replicates.

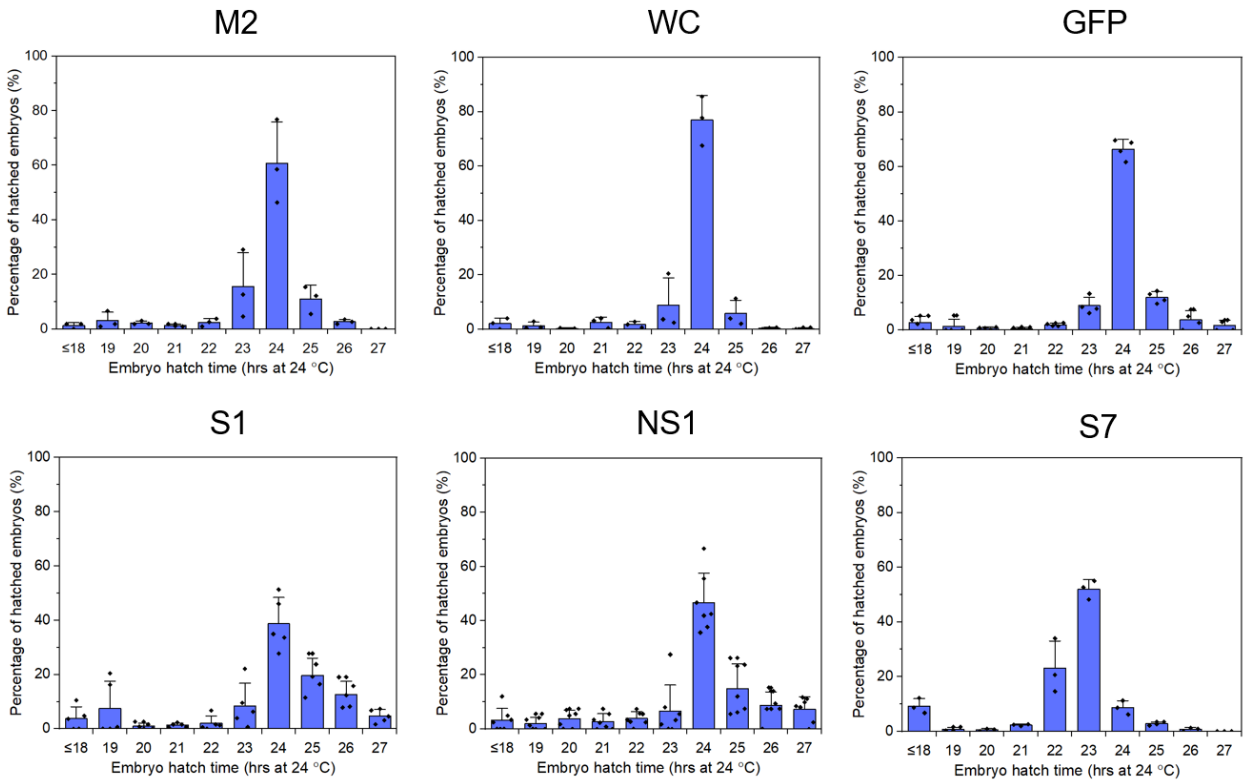

Supplementary Fig. 26. The hatch frequency of embryos incubated at 24 °C. 1 hour embryo collection from various strains were tested. Some strains (i.e., M2, WC, GFP) have a narrow distribution of embryo hatch time, indicating that embryo stage uniformity is high therefore potential higher post cryopreservation survival. Some strains (i.e., S1, NS1) have a broad distribution of embryo hatch time. Some strain hatched earlier (i.e., S7). n=3 or 5 independent replicates. Error bars represent standard deviation.

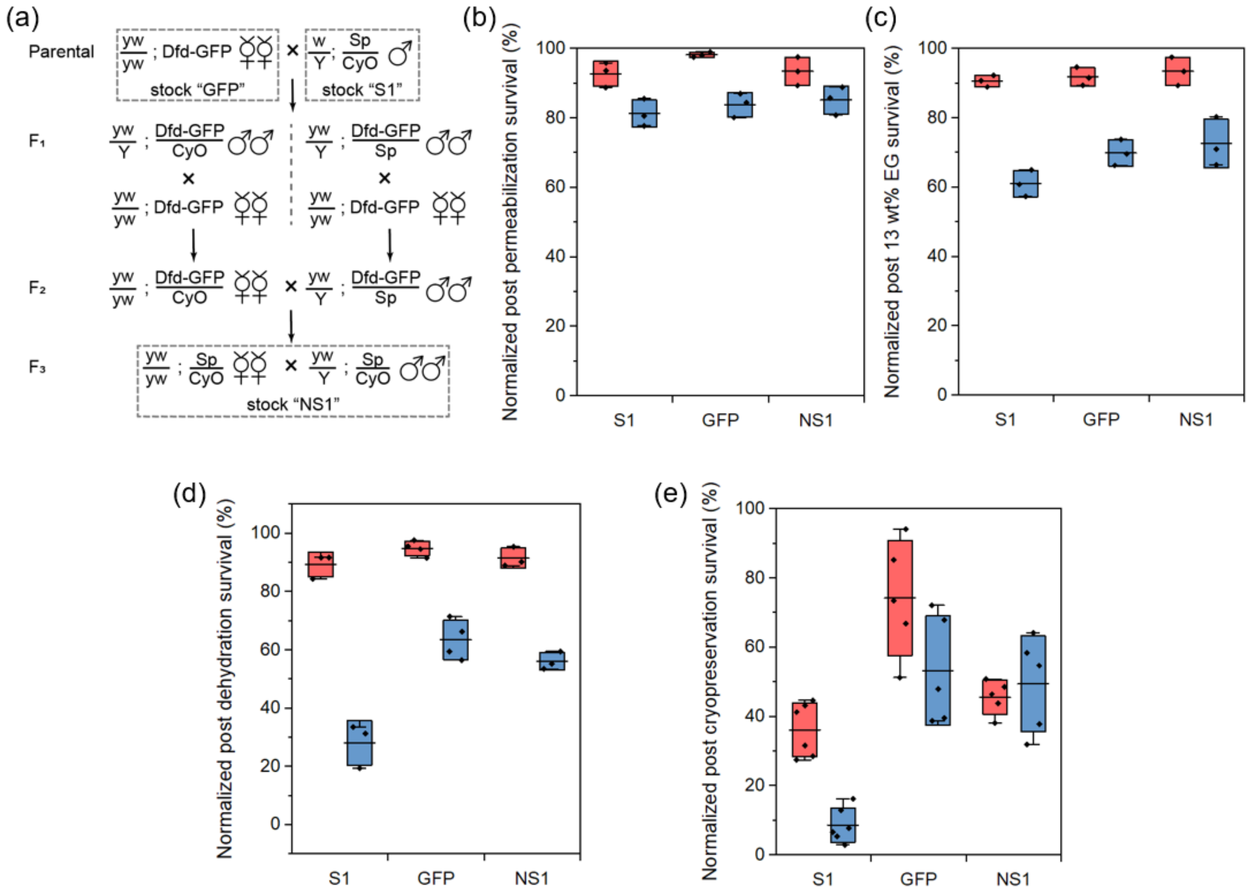

Supplementary Fig. 27. Crossing scheme for NS1 and stepwise survival of S1, NS1 and GFP during cryopreservation. a, Crossing scheme for NS1. **b,** Normalized post permeabilization survival of S1, GFP and NS1. **c,** Normalized post 13 wt% EG treatment survival of S1, GFP and NS1. **d,** Normalized post dehydration survival of S1, GFP and NS1. **e,** Normalized post cryopreservation survival of S1, GFP and NS1. Box and horizontal line represent standard deviation and mean respectively; whiskers represent max and min. n=3 independent replicates for (b-c), n=3 or 4 independent replicates for (d), n=5 or 6 independent replicates for (e). In (b), for S1, 823 embryos were pooled over n=3 independent replicates; for GFP, 882 embryos were pooled over n=3 independent replicates; for NS1, 911 embryos were pooled over n=3 independent replicates. In (c), for S1, 881 embryos were pooled over n=3 independent replicates; for GFP, 926 embryos were pooled over n=3 independent replicates; for NS1, 840 embryos were pooled over n=3 independent replicates. In (d), for S1, 843 embryos were pooled over n=3 independent replicates; for GFP, 1551 embryos were pooled over n=4 independent replicates; for NS1, 829 embryos were pooled over n=3 independent replicates. In (e), for S1, 1817 embryos were pooled over n=6 independent replicates; for GFP, 1859 embryos were pooled over n=5 independent replicates; for NS1, 1478 embryos were pooled over n=5 independent replicates.

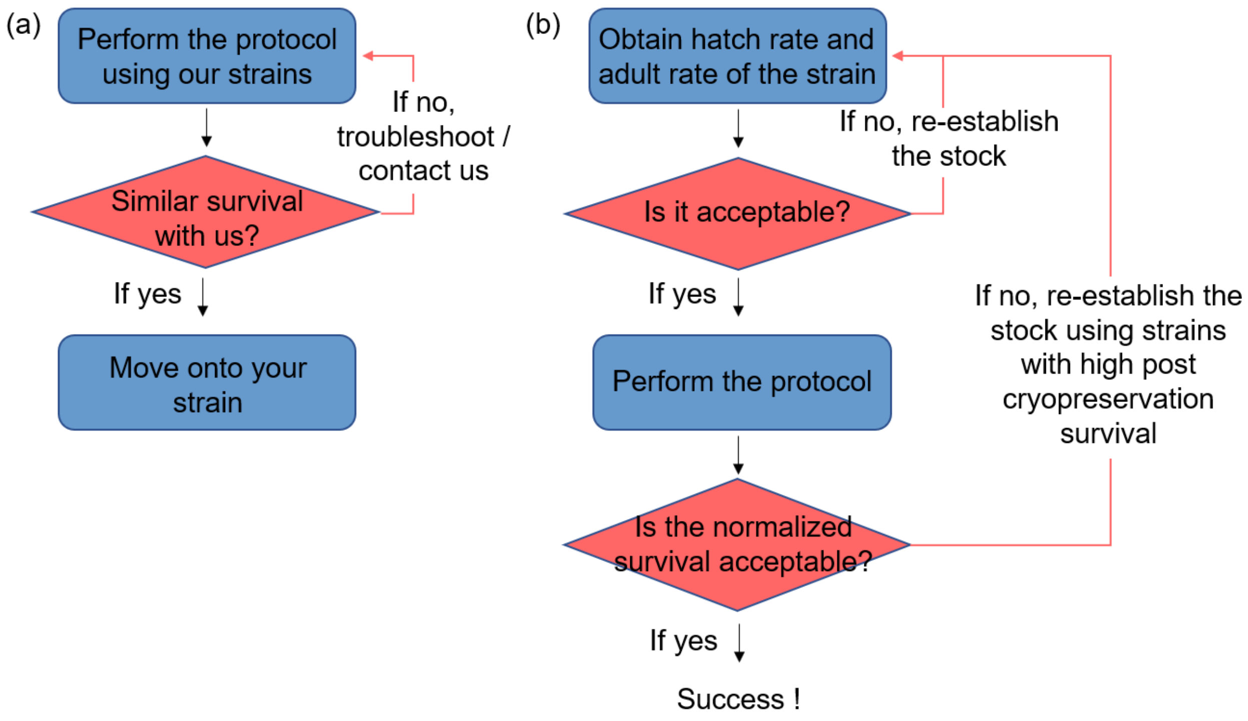

Supplementary Fig. 28. Suggested flowchart for testing our cryopreservation protocol in *Drosophila* labs and stock centers. a, Practice run of the protocol using one of our high survival strains. This step is optional but will provide a good benchmark. **b,** Adoption of our protocol for new strains in other labs. If normalized post cryopreservation survival is low, we suggest performing the protocol with one of our strains first to validate the protocol operation. If that doesn't solve the problem, we suggest outcrossing stock to genetic background that supports high survival post cryopreservation to address the potential genetic background issue.

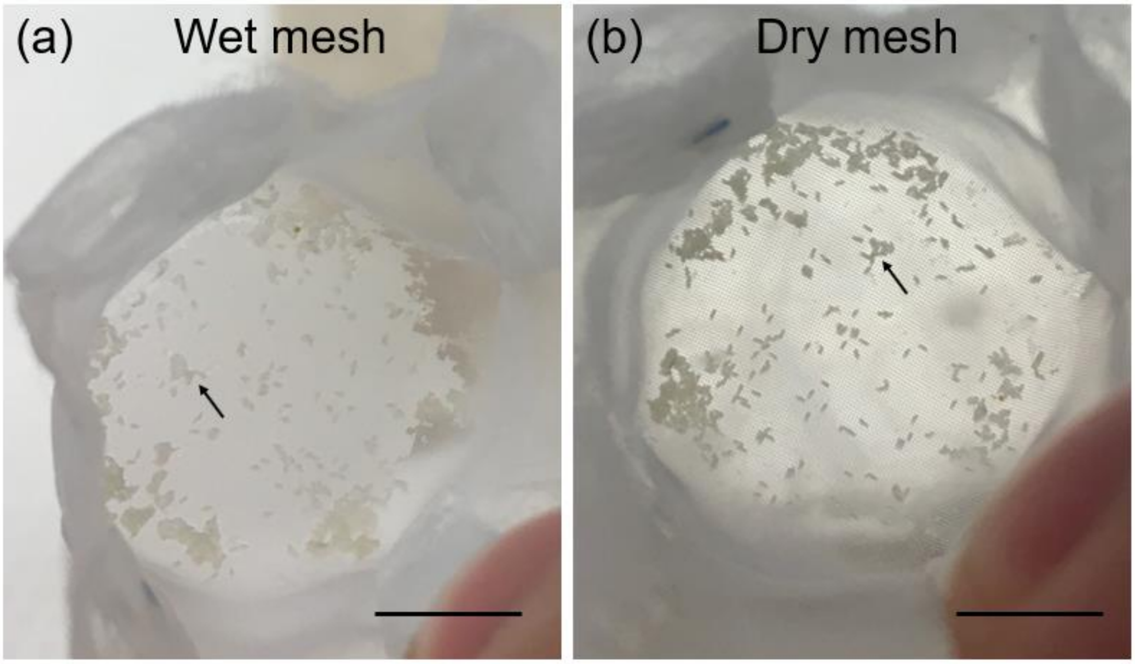

Supplementary Fig. 29. Examples of wet mesh and dry mesh after a dip step in isopropanol. a, A wet mesh can be identified by cloudiness visible to the naked eye indicating liquid at the bottom of the mesh basket. **b,** A dry mesh is recognized due to increased transparency as evidenced by the ability to see through the mesh. The arrows indicate embryos. Scale bar is 1 cm.

Supplementary Table 1. Comparison with previous publications on cryopreservation of *Drosophila melanogaster* embryos

		Mazur et al	Steponkus et al	This work
**Outcome**	*Post cryopreservation survival using wild type*	Hatch rate: 68%<br>Adult rate: 40%	Hatch rate: 83%<br>Adult rate: 54%	Hatch rate[a]: 88%<br>Adult rate: 36%
	Multi-generation cryopreservation?	×	×	✓
	Long term storage?	×	×	✓
	Repeated by non-specialist?	×	×	✓
	Test other mutant strains?	×	×	✓
	Confirm mutation remained?	×	×	✓
**Key procedure**	*Embryo staging method*	morphology	incubation temperature	morphology + incubation temperature
	Embryo incubation temperature	Combination of 24 °C and 17 °C	25 °C	20.1 ± 0.05 °C
	Permeabilization	Specialized device and poor repeatability	Poor repeatability	Simple device and good repeatability
	Cryogen used	Slush nitrogen	Slush nitrogen	Liquid nitrogen
	Device to hold embryos for cryopreservation	Polycarbonate filter	EM copper grid	Nylon mesh
	CPA solution around embryo before vitrification?	Yes	Yes	Minimal
	Specialized device?	Permeabilization setup and slush nitrogen maker	slush nitrogen maker	None
	Post cryopreservation embryo culture method	Placed on agar	Immersed in oil	Floating on medium
**Reference**		1,2	3,4	

[a] *w[1118]* was used as the wildtype

Supplementary Table 2. Comparison of cryomesh with traditional vitrification tools

Device	Sample volume [a] (μL)	# of embryos /run [b]	CPA solution included in the sample?	Cooling rate (°C/min) [c]	Warming rate (°C/min) [d]	Reference
Cryotop	0.1	2-3	yes	~ 69,000	~ 117,000	5
Copper grid	~ 1	~25	yes	~ 24,000	~ 25,000	3,6
Open pulled straw	~ 2	~50	yes	~ 15,000	~ 40,000	7
Quartz capillary	~ 2	~50	yes	~ 30,000	~ 30,000	7
Traditional straw [e]	500	~12,500	yes	~ 1,384	~ 896	3
Cryomesh	NA [f]	>1,700	no	~ 59,600	~ 280,000	This work

[a] This volume includes CPA solution and biomaterials to be cryopreserved unless otherwise noted.

[b] Estimated value based on previous publication. ~ 25 *Drosophila* embryos per microliter was reported in previous publication using the copper grid.[4]

[c] Cooling by plunging into liquid nitrogen.

[d] Warming by convective method.

[e] Devitrification occurs during rewarming, leading to low warming rate.

[f] This is the actual volume of cryopreserve biomaterials themselves, for example, the volume of one dehydrated embryo is estimated to be 3.6 nL (see calculation under "Warming rate modeling" in the Supplementary Materials). More than 1700 *Drosophila* embryos can be placed on one cryomesh (2cm * 2cm) in a monolayer.

Supplementary Table 3. Overview of optimized variables in the cryopreservation procedures

Variable name	Tested conditions*
Age of flies used for embryo collection	1 - 4 days; 9 – 12 days
Embryo stage/age in 20°C incubator	20 hrs; 21 hrs; 22 hrs; 23 hrs; 24 hrs
Soaking time in D-limonene & heptane solution for permeabilization	5 s; 10 s; 20 s; 30 s
Dehydration CPA concentration	33% EG; 43% EG; 39% EG + 9% sorbitol; 35% EG + 17% sorbitol; 53% EG. All units are wt%
Dehydration time	3 min; 9 min; 15 min; 21 min
CPA and/or cocktails	EG; PG; DMSO; EG+PG; EG+DMSO; PG+DMSO
Carrier solution to prepare CPA and unloading solution	Cryobuffer; Schneider medium
Cryogen	Liquid nitrogen; slush nitrogen
Removing CPA around embryos on cryomesh before cooling ?	Yes; no
CPA unloading method	Direct unloading; step unloading
Post cryopreservation embryo culture method	Float on Schneider medium; float on cryobuffer; placed on agar

* Optimal conditions are highlighted in red

Supplementary Table 4. Normalized post cryopreservation survival of 25 different *Drosophila* strains using the same protocol *

Strain info				Post cryopreservation		
Strain name	Description & Genotype	Hatch rate (%)	Adult rate (%)	Normalized hatch rate (%)	Normalized adult rate (%)	Normalized embryo to adult rate (%)
OR	*Oregon-R*	91	77	68.6 ± 5.1	73.2 ± 8.9	50.1 ± 6.1
WC	*w[1118]*	96	83	88 ± 4	36.4 ± 6	32 ± 5.5
WC1	*w[1118]* derivative; outcrossed to isogenize a single X chr. from *w[1118]*	94	89	76.9 ± 8.8	44.4 ± 7.2	33.9 ± 5.7
WC1.1	*w[1118]* derivative; new stock from outcross of WC1, only X chr. of WC1 is maintained	95	60	52.3 ± 2.5	38.8 ± 4.4	20.3 ± 2.1
WC1b	*w[1118]* derivative; 2nd independent stock from outcross of *w[1118]* to isogenize for single X chr.	84	74	71.3 ± 8.9	53 ± 5	40.4 ± 2.7
WC2	*w[1118]* derivative; outcrossed to isogenize a single 2nd chr.	91	85	62.1 ± 10.1	17.3 ± 4	10.6 ± 2.7
WC3	*w[1118]* derivative; outcross to isogenized a single 3rd chr.	93	89	72.2 ± 8.4	35 ± 9.5	25.4 ± 6.9
WC3b	*w[1118]* derivative; 2nd independent stock from outcross of *w[1118]* to isogenize a single 3rd chr.	56	58	83.6 ± 15.4	64.4 ± 16.1	49.2 ± 8.8
GFP [a]	*y[1] w[*]; PBac{y[+mDint2] w[+mC]=Dfd-EGFP.S}VK00037*	62	81	74.3 ± 14.8	53.3 ± 14.1	37.8 ± 5.6
M2	*w[1118]* derivative with a T to G SNP at the position of bp568 in coding sequences of CG1938	97	89	54.5 ± 6.5	35.7 ± 5.9	19.7 ± 5.2
M2-3b	*w[1118]* derivative; outcrossed to isogenize a single 3rd chromosome from M2	81	73	58 ± 6.7	50.4 ± 7.5	29.2 ± 6
S1	*w; Sp/CyO*	68	50	36.2 ± 7.1	8.7 ± 4.5	3 ± 1.5
S2	*po ros/w, FM6*	67	51	51.2 ± 5.6	22.8 ± 7.5	11.9 ± 4.9
S3	*Dhc64C$^{6-12}$, P{neoFRT}80B/TM3*	46	61	53.6 ± 8	46.3 ± 11.4	24.4 ± 5.4
S4	*w[1118]; Sp/CyO; TM2/TM6*	21	44	52.2 ± 6.6	35 ± 4.6	18.5 ± 4.6
S5	*elav-ANFGFP; TM3/TM6*	56	61	40.5 ± 5.1	33.3 ± 2.6	13.6 ± 2.3
S6	*Sp-EM6/FM7-GFP*	74	84	75.8 ± 7.1	17.2 ± 2.4	12.9 ± 1.3
S7	*DhcGFP11-3/TM3 Sb*	64	61	54.1 ± 3.2	32.9 ± 3.5	17.7 ± 2.3
S8	*X; TM3 Sb/TM6B Tb*	36	74	52.1 ± 9.3	26.8 ± 10.8	13.4 ± 3.8
S9	*w; B1[1]/CyO; TM2/TM6, UAS-GAL80*	24	54	45.1 ± 8	20.6 ± 3.8	9.6 ± 3.5
S10	*w; B1[1]/CyO; TM2/TM6*	22	44	48.6 ± 6.5	26.2 ± 11.8	12 ± 3.8
S11 [b]	*bActβ80/UAC-D-GFP*	56	58	67.1 ± 5.7	34.6 ± 6.6	23.6 ± 6.2
S12	*po ros/w, FM6; Sp/CyO*	50	44	41.4 ± 5.4	22 ± 7	9.1 ± 3.3
NS1	*y[1] w[*]; Sp/CyO* (X from strain GFP)	49	57	48.9 ± 3.6	49.4 ± 12.4	22.2 ± 4.9
yw1	*y[1]w[*]* X from strain GFP	89	90	51.2 ± 5.6	40.1 ± 9.4	20.1 ± 2.8

chr. = chromosome; SNP= single nucleotide polymorphism

* normalized survival = survival post cryopreservation / survival without any treatment. n=3-8 independent replicates.

[a] Obtained from Bloomington *Drosophila* Stock Center, BDSC stock number is 30877.

[b] Obtained from Dr. Michael O'Connor's lab.

References

1 Cole, K. W. S., P.D.; Mahowald, A.P. & Mazur, P. . Procedure for the permeabilization and cryobiological preservation of Drosophila embryos. *https://digital.library.unt.edu/ark:/67531/metadc682498/* **May 6** (1993).

2 Mazur, P., Cole, K. W., Hall, J. W., Schreuders, P. D. & Mahowald, A. P. Cryobiological preservation of Drosophila embryos. *Science* **258**, 1932-1935 (1992).

3 Steponkus, P. *et al.* Cryopreservation of Drosophila melanogaster embryos. *Nature* **345**, 170-172 (1990).

4 Steponkus, P. & Caldwell, S. Optimized procedure for the cryopreservation of Drosophila melanogaster embryos. *Cryo-letters* (1993).

5 Jin, B., Kleinhans, F. & Mazur, P. Survivals of mouse oocytes approach 100% after vitrification in 3-fold diluted media and ultra-rapid warming by an IR laser pulse. *Cryobiology* **68**, 419-430 (2014).

6 Mazur, P., Cole, K. W., Schreuders, P. D. & Mahowald, A. P. Contributions of cooling and warming rate and developmental stage to the survival of Drosophila embryos cooled to-205 C. *Cryobiology* **30**, 45-73 (1993).

7 Risco, R., Elmoazzen, H., Doughty, M., He, X. & Toner, M. Thermal performance of quartz capillaries for vitrification. *Cryobiology* **55**, 222-229 (2007).
