# Supplementary material for: Cryopreservation method for Drosophila melanogaster embryos
Source: Nat Commun. 2021 Apr 23;12:2412. doi: 10.1038/s41467-021-22694-z (PMC8065140; doi:10.1038/s41467-021-22694-z)
Supplement: Supplementary file 3 — Reporting Summary [file 41467_2021_22694_MOESM3_ESM.pdf]

nature research

Corresponding author(s): Thomas Hay, John Bischof

Last updated by author(s): 2021/3/5

Reporting Summary

Nature Research wishes to improve the reproducibility of the work that we publish. This form provides structure for consistency and transparency in reporting. For further information on Nature Research policies, see our Editorial Policies and the Editorial Policy Checklist.

Statistics

For all statistical analyses, confirm that the following items are present in the figure legend, table legend, main text, or Methods section.

n/a	Confirmed	
☐	☒	The exact sample size (n) for each experimental group/condition, given as a discrete number and unit of measurement
☐	☒	A statement on whether measurements were taken from distinct samples or whether the same sample was measured repeatedly
☐	☒	The statistical test(s) used AND whether they are one- or two-sided<br>*Only common tests should be described solely by name; describe more complex techniques in the Methods section.*
☐	☒	A description of all covariates tested
☐	☒	A description of any assumptions or corrections, such as tests of normality and adjustment for multiple comparisons
☐	☒	A full description of the statistical parameters including central tendency (e.g. means) or other basic estimates (e.g. regression coefficient) AND variation (e.g. standard deviation) or associated estimates of uncertainty (e.g. confidence intervals)
☐	☒	For null hypothesis testing, the test statistic (e.g. F, t, r) with confidence intervals, effect sizes, degrees of freedom and P value noted<br>*Give P values as exact values whenever suitable.*
☒	☐	For Bayesian analysis, information on the choice of priors and Markov chain Monte Carlo settings
☒	☐	For hierarchical and complex designs, identification of the appropriate level for tests and full reporting of outcomes
☒	☐	Estimates of effect sizes (e.g. Cohen's d, Pearson's r), indicating how they were calculated

Our web collection on statistics for biologists contains articles on many of the points above.

Software and code

Policy information about availability of computer code

Data collection	COMSOL 5.4 was used to collect the numeric simulation data
Data analysis	Microsoft Excel 2016, Origin 2018, SPSS Statistics 23, ImageJ 1.52a were used to analyze the data

For manuscripts utilizing custom algorithms or software that are central to the research but not yet described in published literature, software must be made available to editors and reviewers. We strongly encourage code deposition in a community repository (e.g. GitHub). See the Nature Research guidelines for submitting code & software for further information.

Data

Policy information about availability of data

All manuscripts must include a data availability statement. This statement should provide the following information, where applicable:
- Accession codes, unique identifiers, or web links for publicly available datasets
- A list of figures that have associated raw data
- A description of any restrictions on data availability

The raw data associated with all figures is available upon reasonable request from the corresponding authors (T.H., J.B.).

Field-specific reporting

Life sciences study design

All studies must disclose on these points even when the disclosure is negative.

Sample size	No sample-size calculation was performed. For each data point presents in the paper, 200 - 600 Drosophila embryos were used. The number of replicate is selected such that >1500 embryos were tested to reach a sufficient sample size for each treatment group. Similar sample sizes are used in the literature for similar studies such as "Cole, K.W.S., P.D.; Mahowald, A.P. & Mazur, P. Procedure for the permeabilization and cryobiological preservation of Drosophila embryos. (1993)."
Data exclusions	No data was excluded from the analysis
Replication	The number of replication in each experiment was represented by the number of data points in the presented figures. More than 3 replicates were performed in each experiment. All our attempts at replication were successful.
Randomization	Our sample were allocated randomly
Blinding	For each treatment group, the embryos were randomly selected and the post treatment survival was determined by counting the embryos, larvae and adults. Therefore the bias is minimized and the investigators were not blinded to the different treatment groups of the Drosophila embryos. The investigators were blinded to the genotype of the 25 distinct Drosophila strains.

Reporting for specific materials, systems and methods

We require information from authors about some types of materials, experimental systems and methods used in many studies. Here, indicate whether each material, system or method listed is relevant to your study. If you are not sure if a list item applies to your research, read the appropriate section before selecting a response.

Materials & experimental systems

n/a	Involved in the study	
☒	☐	Antibodies
☒	☐	Eukaryotic cell lines
☒	☐	Palaeontology and archaeology
☐	☒	Animals and other organisms
☒	☐	Human research participants
☒	☐	Clinical data
☒	☐	Dual use research of concern

Methods

n/a	Involved in the study	
☒	☐	ChIP-seq
☒	☐	Flow cytometry
☒	☐	MRI-based neuroimaging

Animals and other organisms

Policy information about studies involving animals; ARRIVE guidelines recommended for reporting animal research

Laboratory animals

Drosophila melanogaster of 25 distinct strains were used. The strain information was provided in the Methods under the section of 'Stock maintenance'. 1-15 days old male and female flies were used.
Designation and Genotype information (i.e., in parenthesis) are listed as below:
OR (Oregon-R)
WC (w[1118])
WC1 (w[1118] derivative; outcrossed to isogenize a single X chromosome from w[1118])
WC1b (w[1118] derivative; 2nd independent stock from outcross of w[1118] to isogenize for single X chromosome)
WC1.1 (w[1118] derivative; new stock from outcross of WC1, only X chromosome of WC1 is maintained)
WC2 (w[1118] derivative; outcrossed to isogenize a single 2nd chromosome)
WC3 (w[1118] derivative; outcrossed to isogenize a single 3rd chromosome)
WC3b (w[1118] derivative; 2nd independent stock from outcross of w[1118] to isogenize for single 3rd chromosome)
GFP (y[1] w[*]; PBac{y[+mDint2] w[+mC]=Dfd-EGFP.S}VK00037) BDSC # 30877.
M2 (w[1118] derivative with a T to G SNP at the position of bp568 in coding sequences of CG1938)
M2-3b (w[1118] derivative; outcrossed to isogenize a single 3rd chromosome from M2)
S1 (w; Sp/CyO)
S2 (po ros/w FM6)
S3 (Dhc64C6-12, P{neoFRT}80B/TM3)
S4 (w[1118]; Sp/CyO; TM2/TM6)
S5 (elav-GAL4 ANF-GFP; TM3/TM6)
S6 (Sp-EM6/FM7-GFP)
S7 (DhcGFP11-3/TM3 Sb)
S8 (TM3 Sb/TM6B Tb)
S9 (w; Bl[1]/CyO; TM2/TM6 UAS-GAL80)
S10 (w; Bl[1]/CyO; TM2/TM6)
S11 (bActβ80/UAC-D-GFP)

	S12 (po ros/w FM6; Sp/CyO)<br>NS1 (y[1] w[*]; Sp/CyO)<br>yw1 (y[1] w[*])
Wild animals	no wild animals were used in this study.
Field-collected samples	no field-collected samples were used in this study
Ethics oversight	no ethical oversight is required in this study

Note that full information on the approval of the study protocol must also be provided in the manuscript.
